# Survey of vector-borne agents in feral cats and first report of *Babesia gibsoni* in cats on St Kitts, West Indies

**DOI:** 10.1186/s12917-017-1230-1

**Published:** 2017-11-13

**Authors:** Patrick John Kelly, Liza Köster, Jing Li, Jilei Zhang, Ke Huang, Gillian Carmichael Branford, Silvia Marchi, Michel Vandenplas, Chengming Wang

**Affiliations:** 1 One Health Center for Zoonoses and Tropical Veterinary Medicine, Ross University School of Veterinary Medicine, P.O. Box 334, Basseterre, St. Kitts, Saint Kitts and Nevis; 2 Glasgow University School of Veterinary Medicine, Small Animal Hospital, Garscube Campus, 464 Bearsden Road, Glasgow, G61 1QH UK; 3 College of Veterinary Medicine, Yangzhou University, Yangzhou, Jiangsu China; 4 College of Veterinary Medicine, Auburn University, Auburn, AL USA

**Keywords:** *Babesia*, Cat, *Ehrlichia*, *Rickettsia*, Vector-borne

## Abstract

**Background:**

As there is little data on vector-borne diseases of cats in the Caribbean region and even around the world, we tested feral cats from St Kitts by PCR to detect infections with *Babesia*, *Ehrlichia* and spotted fever group *Rickettsia* (SFGR) and surveyed them for antibodies to *Rickettsia rickettsii* and *Ehrlichia canis*.

**Results:**

Whole blood was collected from apparently healthy feral cats during spay/ neuter campaigns on St Kitts in 2011 (*N* = 68) and 2014 (*N* = 52). Sera from the 52 cats from 2014 were used to detect antibodies to *Ehrlichia canis* and *Rickettsia rickettsii* using indirect fluorescent antibody tests and DNA extracted from whole blood of a total of 119 cats (68 from 2011, and 51 from 2014) was used for PCRs for *Babesia*, *Ehrlichia* and *Rickettsia*. We could not amplify DNA of SFG *Rickettsia* in any of the samples but found DNA of *E. canis* in 5% (6/119), *Babesia vogeli* in 13% (15/119), *Babesia gibsoni* in 4% (5/119), mixed infections with *B. gibsoni* and *B. vogeli* in 3% (3/119), and a poorly characterized *Babesia* sp. in 1% (1/119). Overall, 10% of the 52 cats we tested by IFA for *E. canis* were positive while 42% we tested by indirect fluorescent antibody (IFA) for *R. rickettsii* antigens were positive.

**Conclusions:**

Our study provides the first evidence that cats can be infected with *B. gibsoni* and also indicates that cats in the Caribbean may be commonly exposed to other vector-borne agents including SFGR*, E. canis* and *B. vogeli*. Animal health workers should be alerted to the possibility of clinical infections in their patients while public health workers should be alerted to the possibility that zoonotic SFGR are likely circulating in the region.

## Background

Feral cats are common on Caribbean islands in the West Indies where they are valued by local residents due to their role in controlling rodents and rodent-associated diseases [1]. While feral cats in the region are known to be commonly infected with external and internal parasites [2–5], haemoplasmas [6] and feline immunodeficiency virus [7–9], there is very little data on vector-borne agents. Although studies on dogs have shown vector-borne diseases are very common in the Caribbean region [10–13], there have been only few studies on these infections in cats. *Bartonella* spp. have been shown to occur on three Caribbean islands [7, 9, 14, 15], cats seropositive against *Rickettsia rickettsii* have been identified on St Kitts [16], and DNA of *Ehrlichia canis* and *Babesia vogeli* have been found in cats in Trinidad [6]. As studies from southern Africa [17], China [18], Italy [19], Japan [20], Portugal [21], Spain [22], Tasmania [23], and the United States of America [24] have shown cats can be infected with a number of vector-borne agents, we carried out a serology and PCR survey to determine exposure of cats on St Kitts to the more important vector-borne agents, mainly *Ehrlichia*, *Babesia* and spotted fever group *Rickettsia* (SFGR).

## Methods

### Animals

This study was approved by the Institutional Animal Care and Use Committee of Ross University School of veterinary Medicine (RUSVM).

The Feral Cat Project (FCP) of RUSVM traps, neuters or spays, and releases feral cats on St Kitts as a welfare and disease control initiative. Whole blood was collected from a convenience sample of 52 cats trapped in and around Basseterre, the capital of the island, between September and November 2014. Although no blood work was performed on the cats, all appeared normal on physical examination and during the 3 to 4 days they were in captivity. Immediately following collection, sera were separated and stored at −80 °C until serology was performed. For PCR, the buffy coat and superficial erythrocyte layers of centrifuged ETDA whole blood were collected and frozen at −80 °C until thawed for DNA extraction as described below. One cell sample was lost meaning there were 52 sera available for analysis and 51 DNA samples.

We also used archived DNA which had been extracted from buffy coats and superficial erythrocytes collected from 68 feral cats trapped and neutered as part of the FCP in 2011. Sera were not available from these cats. As above, although no routine laboratory health screens were performed, these cats also appeared healthy on physical examination and during their captivity.

### Indirect fluorescent antibody assay

Indirect fluorescent antibody (IFA) testing was performed using *E. canis* (Oklahoma strain) and *R. rickettsii* (both kindly supplied by Dr. G Dasch, Centers for Disease Control, Georgia, Atlanta, USA) and commercial fluorescein isothiocyanate-conjugated anti-cat IgG (Kirkegaard & Perry Laboratories) as described previously [18, 25]. Sera were initially screened at a 1:80 dilution in PBS (pH 7.4) and positive reactors were examined again at a 1:640 dilution.

### DNA extraction

The DNA was extracted from aliquots (200 μL) of buffy coats using the QIAamp DNA Blood Mini Kit (QIAGEN, Valencia, CA, USA) according to the manufacturer’s instructions. The DNA was eluted in 200 μL elution buffer and shipped to Yangzhou University College of Veterinary Medicine of Jiangsu province, China at room temperature where it was frozen at −80 °C until PCRs were performed.

### PCRs

A conventional PCR was used as described previously [26] to detect DNA of SFGR using primers *ompB*-forward (5′-CGACGTTAACGGTTTCTCATTCT-3′) and *ompB*-reverse (5′-ACCGGTTTCTTTGTAGTTTTCGTC-3′) that amplify a 252 bp portion of the outer membrane protein B.

The *Ehrlichia* FRET-PCR [27] and pan-*Babesia* FRET-PCR [28] used in this study were performed in a LightCycler 480-II real-time PCR platform as described before. The *Ehrlichia* FRET-PCR amplifies a 210 bp fragment of the *16S rRNA* and can detect the five well recognized *Ehrlichia* species with a detection sensitivity of 5 copies per PCR reaction [27].

The *Babesia* spp. FRET-PCR amplifies a 282 to 293 bp segment of the *18S rRNA* of 22 *Babesia* spp. with of a sensitivity of as low as 2 copies of the *18S rRNA* per reaction [28]. To further confirm the identification of *Babesia* species, species-specific PCRs for *B. vogeli* (upstream primer: 5′-TTHGCGATGKWACCATTCAAGTTTCTG-3′; downstream primer: 5′-CCCAACCGTTCCTATTAACCATTACT-3′) and *B. gibsoni* (upstream primer: 5′-TTHGCGATGKWACCATTCAAGTTTCTG-3′; downstream primer 5′-CGTTCCTATTAACCATTACTAAGGTTCACA-3′) were established which targeted a hyper-variable region of the *18S rRNA* (about 540 bp). These PCRs were performed under the same conditions as described above for the *Babesia* spp. FRET-qPCR.

All PCR products obtained were further verified by electrophoresis through 2% agarose gels (BIOWEST1, Hong Kong, China) before being purified using the QIAquick PCR Purification Kit (Qiagen), and sent for sequencing with forward and reverse primers (BGI, Shanghai, China).

### Phylogenetic analysis

**Fig. 1:**
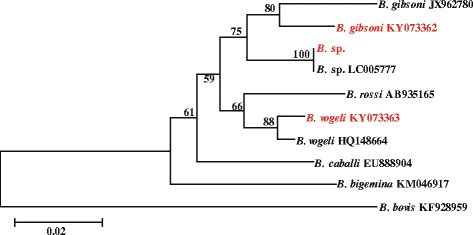
Phylogeny of *18S rRNA* of *Babesia* species. The variable region of the *18S rRNA* (540 bp) of *Babesia* strains identified in this study (in red font) are compared with those of other *Babesia* sequences deposited in GenBank (in black font). Branch lengths are measured in nucleotide substitutions and numbers show branching percentages in bootstrap replicates. Scale bar represents the percent sequence diversity

Phylogenetic analysis was performed based on the variable region of the *Babesia* 18S rRNA gene. Sequences identified in this study and obtained from GenBank were aligned using the Clustalx 1.83 alignment software. Based on these alignments, phylogenetic trees were constructed by the neighbor-joining method using the Kimura 2-parameter model with MEGA 6.0. Bootstrap values were calculated using 500 replicates (Fig 1).

## Results

### PCRs for Rickettsia, Ehrlichia and Babesia

The PCRs for SFGR were negative with DNA extracted from the 68 feral cats trapped in 2011 and the 51 cats trapped in 2014 (Table 1). Although all 68 DNA samples from cats trapped in 2011 were negative in the *Ehrlichia* FRET-PCR, six of 51 samples (12%) from 2014 were positive. Sequencing of the amplicons of the positive PCRs showed all had identical sequences with 28 *E. canis* strains in GenBank. Five of the samples were from cats that were positive by IFA for antibodies to *E. canis* and one cat was seronegative.

**Table 1:** Serology and PCR results for blood samples collected from feral cats on St Kitts in 2011 and 2014

Collection date	Test performed	% positive (N)	Species identified
2011	*Ehrlichia* FRET-PCR	0% (0/68)	None
	*Babesia* FRET-PCR	32% (22/68)	4 *B. gibsoni* 14 *B. vogeli* 3*B. vogeli* and *B. gibsoni* 1 *Babesia* sp.
	*Rickettsia* PCR	0% (0/68)	Not applicable
2014	IFA for *Ehrlichia*	10% (5/52)	Not applicable
	IFA for SFG *Rickettsia*	42% (22/52)	Not applicable
	*Ehrlichia* FRET-PCR	12% (6/51)	6 *E. canis*
	*Babesia* FRET-PCR	2% (1/51)	1 *B. gibsoni*
	*Rickettsia* PCR	0% (0/51)	Not applicable

Twenty-two of the 68 samples (31%) collected in 2011 were positive in the pan-*Babesia* FRET-PCR while only one of the 51 samples (2%) collected in 2014 was positive. Sequencing of the positive amplicons from the pan-*Babesia* FRET-PCR and those of the specific *B. vogeli* and *B. gibsoni* PCRs revealed one *B. gibsoni*-positive sample in 2014 (Table 1). The 21 positive samples from 2011 were mainly *B. vogeli* (67%; 14/21) with three samples (14%; 3/21) having evidence of a mixed infection with *B. gibsoni* and *B. vogeli* and one sample being a poorly characterized *Babesia* sp. (Fig. 1). The sequences of the amplicons we identified as *B. vogeli* in our study were all identical, as was the case with the amplicons we identified as *B. gibsoni*. They have been deposited in GenBank (*B. vogeli* accession #: KY073363; *B. gibsoni* accession #: KY073362) and are identical to 19 *B. vogeli* and identical to 37 *B. gibsoni* sequences (100% cover and 100% ident) recorded in GenBank, respectively.

### Serology for *Rickettsia* and *Ehrlichia*

Of the 52 stray cats sampled in 2014, only 10% (5/52) had antibodies to *E. canis* in the IFAs and all were at low titer, arbitrarily defined as 1:80 to 1:320. More of these cats were seropositive for SFGR (42%; 22/52) with 5 (10%) having high titers, arbitrarily defined as 1:640 or greater (Table 1).

## Discussion

Our results show feral cats on St Kitts are not uncommonly exposed to a variety of vector-borne agents. In a report from 2010 on feral cats from St Kitts [29], 66% of cats were seropositive for SFGR and our later studies confirm exposure to SFGR is common in cats on the island with 42% of the cat samples collected in 2014 being positive. These levels of SFGR seropositivity are somewhat higher than those reported elsewhere, mainly southern Africa (29%) [17], China (21%) [18], Italy (55%) [19], Japan (1%) [20], Portugal (19%) [21], Spain (28%) [22], Tasmania (59%) [23], and the US (11%-17%) [24, 30]. We suspect this is most likely due to the warm and humid tropical conditions on the island throughout the year which promotes the survival of ticks and fleas which are the main vectors of the SFGR*.*

As there is considerable cross-reactivity between the numerous SFGR in IFA tests [31] we could not determine the species infecting the cats we studied. Although cats have been found to be PCR positive for *Rickettsia conorii* and *Rickettsia masilliae* in Spain [22], animals [32–34] and people [35] infected with SFGR are generally only rickettsemic for very short periods and it was not unexpected that our PCR assays for *Rickettsia* were negative.

A number of SFGR have been shown to be present in ticks and fleas on St Kitts, mainly *Rickettsia felis* [29], *Rickettsia africae* [7], the Israeli tick typhus group rickettsia, *R. rickettsii* and *Rickettsia rhipicephali* [36]. Of these, *R. felis* and *R. africae* are found most commonly but, as *R. africae* is found in *Amblyomma variegatum*, the tropical bont tick, which mainly feeds on large ruminants and only very infrequently on cats, it seems most likely the seroconversions we recorded were due to exposure to *R. felis* which has been found in 19% of cat fleas on the island [29]. *Rickettsia felis* is a recently described SFGR that is an emerging pathogen causing flea-borne spotted fever in people [37]. The cat flea, *Ctenocephalides felis*, is considered to be its major reservoir and biological vector [38]. Cats seem unlikely to be important vertebrate reservoirs [39] as they are rickettsemic for only short periods after infection [40] and, in PCR surveys, they are mostly found to be PCR negative [18, 22, 24] although sometimes PCR positive animals have been reported [41]. There is little information on the pathogenicity of *R. felis* in cats but most infections appear subclinical with a brief rickettsemia before reactive antibodies develop and clear infections [40].

Although the SFGR identified on St Kitts to date, with the exception of *R. rhipicephali*, are human pathogens there is little data on these zoonoses in the Caribbean. Infections with *R. africae* have been described in tourists to the region [42] and a small serosurvey showed 34% of people from 10 islands had serological evidence of a previous infection [43]. Further studies are needed to determine the extent of SFG rickettsioses in the Caribbean and the role cats might play in these infections.

Previous studies have reported a variety of *Babesia* in wild and domestic cats from around the world [44–53]. The poorly characterized *Babesia* sp. we found was most closely related (99.3%; 281/283 matches) to a *Babesia* (KP221651) identified in a sheep from St Kitts (Fig. 1) in a previous study [28]. Further studies are needed to further characterize this organism and identify its vector.

The *Babesia* we identified most commonly in our Caribbean cats was *B. vogeli* which has also been found in apparently healthy cats in Brazil [48, 54], Thailand [46] and Portugal [51]. *B. vogeli* commonly infects dogs in tropical and subtropical areas with prevalences of 4 to 60% [55] and studies in St Kitts have found 7% and 12% of dogs were PCR positive [11, 56]. The organism is transmitted by *Rhipicephalus sanguineus* sensu lato and infections are mostly subclinical in dogs with most infected animals becoming subclinical carriers [16]. Although *R. sanguineus* s. l. is common on St Kitts and in the Caribbean and is essentially the only tick found on dogs in the region [11, 56], it has a near-strict host preference for dogs. The only ectoparasites we identified on the cats we studied were cat fleas (*C. felis*) and the fur mite (*Lynxacarus radovskyi*), both of which were common. We did not find ticks on any of the cats we tested and while there was relatively high prevalence of *B. vogeli* in our study. The reason that ticks were infrequently found on cats is due to that they are such efficient groomers.

To the best of our knowledge, the other *Babesia* we found to occur commonly, *B. gibsoni*, has not previously been described in cats. The organism, however, is encountered relatively frequently (5%) in healthy dogs on St Kitts and also in dogs with a suspected vector-borne disease (15%) and clinical and laboratory abnormalities [11, 56]. The cats we found infected in our study were all apparently normal on physical examination and therefore seem to have had subclinical infections. It is known that infections with other *Babesia*, mainly *B. felis* [55], *B. canis presentii* [44] and *B. lengau* [50], can be associated with laboratory abnormalities and clinical signs and further studies are underway in our laboratories to determine the effects of infections in cats with the *Babesia* we identified in our study.

The third most prevalent vector-borne agent we detected in our cats was *E. canis*. This is an agent of canine monocytic ehrlichiosis which is transmitted by the brown dog tick, *R. sanguineus.* Infections in dogs are very common around the world and on St Kitts infection levels of 12 to 27% have been reported [11, 56]. There are relatively few studies on *E. canis* in cats but seropositive animals have been described from around the world, for example 6% in southern Africa [25], 6 to 45% in Brazil [57], 10% in Spain [58] and 82% in the US [59]. Elsewhere, seroprevalence studies have tended to overestimate infection rates demonstrated by positive PCR [60], but in our study all seropositive animals were also PCR positive. This might indicate the cats in our study were relatively recently infected and had not had time to self-cure as has been shown to occur in dogs [61]. Although all the cats in our study appeared healthy on physical examination, cats infected with *E. canis* have been reported to suffer from fever, lymphadenomegaly, splenomegaly, polyarthritis, bone marrow hypoplasia and anemia [60, 62].

## Conclusions

Our study shows feral cats on St Kitts are relatively commonly exposed and infected with a variety of vector-borne agents. In many cases the effects of infection on cats is unknown and potentially treatable conditions might be going undiagnosed. Also, many of the agents can infect dogs and people which live in close proximity to the cats and share their ectoparasites. Animal health workers should be alerted to the possibility of clinical infections in their feline and canine patients while public health workers should be alerted to the possibility that cats may play a role in the epidemiology of zoonotic vector-borne diseases in the region.
